# 4-(4-Fluoro­anilino)-*N*-(4-fluoro­phen­yl)-3-nitro­benzamide

**DOI:** 10.1107/S1600536810040687

**Published:** 2010-10-23

**Authors:** Yong Wang, Kaiqing Fan, Chenghong Li, Changhua Ge

**Affiliations:** a School of Pharmaceutical and Chemical Engineering, Taizhou University, Linhai 317000, People’s Republic of China; b Agronomy Department, Jiangsu Polytechnic College of Agriculture and Forestry, Jurong 212400 Jiangsu, People’s Republic of China

## Abstract

In the title compound, C_19_H_13_F_2_N_3_O_3_, the anilinobenzamide unit is essentially planar, with a maximum deviation of 0.036 (3) Å. The nitro group and the benzene ring form dihedral angles of 9.6 (5)and 62.20 (8)°, respectively, with the anilinobenzamide unit. An intra­molecular N—H⋯O inter­action occurs. In the crystal, mol­ecules are linked by weak inter­molecular C—H⋯O, N—H⋯O and C—H⋯F hydrogen bonds, which stabilize the structure.

## Related literature

For comparison of bond lengths, see: Allen *et al.* (1987 ▶). For the synthetic procedure, see: Schelz & Inst (1978 ▶).
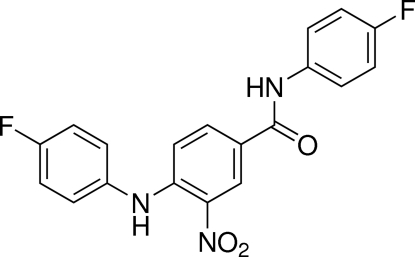


## Experimental

### 

#### Crystal data

C_19_H_13_F_2_N_3_O_3_
*M* *_r_* = 369.32
Triclinic, 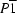

*a* = 7.8510 (16) Å
*b* = 8.2720 (17) Å
*c* = 13.835 (3) Å
α = 74.75 (3)°
β = 85.67 (3)°
γ = 70.76 (3)°
*V* = 818.4 (3) Å^3^
*Z* = 2
Mo *K*α radiation
μ = 0.12 mm^−1^
*T* = 293 K
0.30 × 0.20 × 0.10 mm

#### Data collection

Enraf–Nonius CAD-4 diffractometer
Absorption correction: ψ scan (North *et al.*, 1968 ▶) *T*_min_ = 0.965, *T*_max_ = 0.988
3198 measured reflections
2962 independent reflections
1559 reflections with *I* > 2σ(*I*)
*R*_int_ = 0.026
3 standard reflections every 200 reflections  intensity decay: 1%

#### Refinement

*R*[*F* ^2^ > 2σ(*F* ^2^)] = 0.058
*wR*(*F* ^2^) = 0.155
*S* = 1.00
2962 reflections
245 parameters
H-atom parameters constrained
Δρ_max_ = 0.16 e Å^−3^
Δρ_min_ = −0.18 e Å^−3^

### 

Data collection: *CAD-4 Software* (Enraf–Nonius, 1994) ▶; cell refinement: *CAD-4 Software* ▶; data reduction: *XCAD4* (Harms & Wocadlo, 1995 ▶); program(s) used to solve structure: *SHELXS97* (Sheldrick, 2008 ▶); program(s) used to refine structure: *SHELXL97* (Sheldrick, 2008 ▶); molecular graphics: *PLATON* (Spek, 2009 ▶); software used to prepare material for publication: *SHELXL97*.

## Supplementary Material

Crystal structure: contains datablocks global, I. DOI: 10.1107/S1600536810040687/pv2329sup1.cif

Structure factors: contains datablocks I. DOI: 10.1107/S1600536810040687/pv2329Isup2.hkl

Additional supplementary materials:  crystallographic information; 3D view; checkCIF report

## Figures and Tables

**Table 1:** Hydrogen-bond geometry (Å, °)

*D*—H⋯*A*	*D*—H	H⋯*A*	*D*⋯*A*	*D*—H⋯*A*
N1—H1*A*⋯O3^i^	0.86	2.37	3.185 (4)	158
N2—H2*A*⋯O2	0.86	1.98	2.636 (4)	132
C2—H2*B*⋯O3^i^	0.93	2.40	3.240 (5)	151
C10—H10*A*⋯F1^ii^	0.93	2.53	3.205 (4)	130
C15—H15*A*⋯O1^iii^	0.93	2.55	3.454 (4)	164
C16—H16*A*⋯F2^iv^	0.93	2.39	3.272 (5)	158

Symmetry codes: (i) 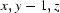; (ii) 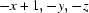; (iii) 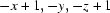; (iv) 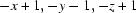.

**Fig. 1.**
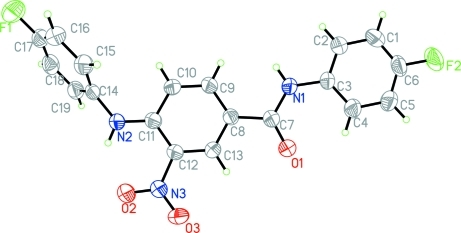
The molecular structure of (I), showing the atom-numbering scheme and displacement ellipsoids at the 30% probability level.

**Fig. 2.**
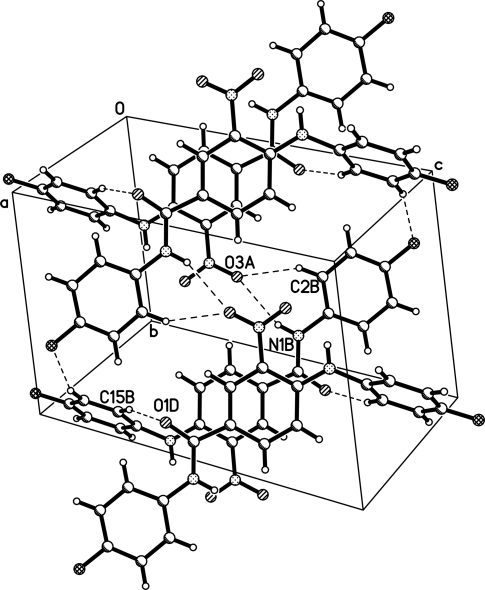
A packing diagram of (I). The intermolecular hydrogen bonds are shown as dashed lines.

## supplementary crystallographic information

### Comment

The crystal structure of the title compound, (I), is presented in this article. In the title molecule (Fig. 1), the bond lengths and angles are within normal ranges (Allen *et al.*, 1987). The phenylaminobenzamide moiety (C1–C13/N1/O1) is essentially planar with maximum deviation of any atom being 0.036 (3) Å for C11 with F2 lying 0.109 (4) Å out of its plane, nitro group (N3/O2/O3) titlted at an angle 9.6 (5)° from its plane and the phenyl ring (C14–C19) inclined at 62.20 (8)° with its plane. In the crystal structure, weak intermolecular C—H···O, N—H···O and C—H···F hydrogen bonds (Table 1) link the molecules (Fig. 2), in which they may be effective in stabilizing the structure.

### Experimental

4-Chloro-3-nitrobenzamide (4.0 g, 0.02 mol) was heated in 4-fluorobenzenamine (10 ml) for 18 h at 403 K. On completion of the reaction (TLC control) was added ethanol (50 ml), at room temperature. The red precipitate thus formed was filtered, washed with cold ethanol (2 × 15 ml), dried over sodium sulfate to provide 5.8 g (79%) of (I) (Schelz & Inst, 1978). The compound (I) was purified by crystallizing from methanol. The crystals of (I) suitable for X-ray diffraction were obstained by slow evaporation of a methanol solution.

### Refinement

H atoms were positioned geometrically, with N—H = 0.86 and C—H = 0.93 Å, and constrained to ride on their parent atoms, with *U*_iso_(H) = 1.2*U*_eq_(C/N).

### Crystal data

**Table:**

C_19_H_13_F_2_N_3_O_3_	*Z* = 2
*M**_r_* = 369.32	*F*(000) = 380
Triclinic, *P*1	*D*_x_ = 1.499 Mg m^−^^3^
Hall symbol: -P 1	Mo *K*α radiation, λ = 0.71073 Å
*a* = 7.8510 (16) Å	Cell parameters from 25 reflections
*b* = 8.2720 (17) Å	θ = 9–12°
*c* = 13.835 (3) Å	µ = 0.12 mm^−^^1^
α = 74.75 (3)°	*T* = 293 K
β = 85.67 (3)°	Block, colourless
γ = 70.76 (3)°	0.30 × 0.20 × 0.10 mm
*V* = 818.4 (3) Å^3^	

### Data collection

**Table:**

Enraf–Nonius CAD-4 diffractometer	1559 reflections with *I* > 2σ(*I*)
Radiation source: fine-focus sealed tube	*R*_int_ = 0.026
graphite	θ_max_ = 25.3°, θ_min_ = 1.5°
ω and 2θ scans	*h* = 0→9
Absorption correction: ψ scan (North *et al.*, 1968)	*k* = −9→9
*T*_min_ = 0.965, *T*_max_ = 0.988	*l* = −16→16
3198 measured reflections	3 standard reflections every 200 reflections
2962 independent reflections	intensity decay: 1%

### Refinement

**Table:**

Refinement on *F*^2^	Secondary atom site location: difference Fourier map
Least-squares matrix: full	Hydrogen site location: inferred from neighbouring sites
*R*[*F*^2^ > 2σ(*F*^2^)] = 0.058	H-atom parameters constrained
*wR*(*F*^2^) = 0.155	*w* = 1/[σ^2^(*F*_o_^2^) + (0.065*P*)^2^] where *P* = (*F*_o_^2^ + 2*F*_c_^2^)/3
*S* = 1.00	(Δ/σ)_max_ < 0.001
2962 reflections	Δρ_max_ = 0.16 e Å^−^^3^
245 parameters	Δρ_min_ = −0.18 e Å^−^^3^
0 restraints	Extinction correction: *SHELXL97* (Sheldrick, 2008), Fc^*^=kFc[1+0.001xFc^2^λ^3^/sin(2θ)]^-1/4^
Primary atom site location: structure-invariant direct methods	Extinction coefficient: 0.021 (4)

### Special details

**Table:**

Geometry. All e.s.d.'s (except the e.s.d. in the dihedral angle between two l.s. planes) are estimated using the full covariance matrix. The cell e.s.d.'s are taken into account individually in the estimation of e.s.d.'s in distances, angles and torsion angles; correlations between e.s.d.'s in cell parameters are only used when they are defined by crystal symmetry. An approximate (isotropic) treatment of cell e.s.d.'s is used for estimating e.s.d.'s involving l.s. planes.
Refinement. Refinement of *F*^2^ against ALL reflections. The weighted *R*-factor *wR* and goodness of fit *S* are based on *F*^2^, conventional *R*-factors *R* are based on *F*, with *F* set to zero for negative *F*^2^. The threshold expression of *F*^2^ > σ(*F*^2^) is used only for calculating *R*-factors(gt) *etc*. and is not relevant to the choice of reflections for refinement. *R*-factors based on *F*^2^ are statistically about twice as large as those based on *F*, and *R*- factors based on ALL data will be even larger.

### Fractional atomic coordinates and isotropic or equivalent isotropic displacement parameters (Å^2^)

**Table:**

	*x*	*y*	*z*	*U*_iso_*/*U*_eq_	
F1	0.7511 (3)	0.0655 (3)	−0.12706 (14)	0.0823 (8)	
N1	0.1978 (4)	−0.2157 (3)	0.58538 (17)	0.0468 (7)	
H1A	0.2383	−0.2652	0.5372	0.056*	
F2	−0.0468 (3)	−0.6359 (3)	0.90592 (15)	0.0839 (8)	
C1	0.0848 (6)	−0.5958 (5)	0.7467 (3)	0.0687 (12)	
H1C	0.0888	−0.7083	0.7443	0.082*	
N2	0.4401 (4)	0.3033 (3)	0.20096 (18)	0.0511 (8)	
H2A	0.4232	0.4141	0.1934	0.061*	
O1	0.1462 (4)	0.0382 (3)	0.63333 (16)	0.0645 (8)	
C2	0.1458 (5)	−0.4877 (4)	0.6679 (3)	0.0618 (11)	
H2B	0.1932	−0.5290	0.6120	0.074*	
O2	0.3708 (4)	0.5620 (3)	0.29073 (19)	0.0787 (9)	
C3	0.1378 (4)	−0.3197 (4)	0.6703 (2)	0.0424 (8)	
N3	0.3373 (4)	0.4670 (4)	0.3685 (2)	0.0579 (9)	
C4	0.0683 (5)	−0.2610 (5)	0.7541 (2)	0.0515 (9)	
H4A	0.0612	−0.1478	0.7569	0.062*	
O3	0.3066 (5)	0.5176 (4)	0.4448 (2)	0.1081 (13)	
C5	0.0093 (5)	−0.3689 (5)	0.8339 (2)	0.0573 (10)	
H5A	−0.0360	−0.3301	0.8909	0.069*	
C6	0.0189 (5)	−0.5329 (5)	0.8276 (3)	0.0577 (10)	
C7	0.1996 (4)	−0.0465 (4)	0.5702 (2)	0.0413 (8)	
C8	0.2697 (4)	0.0325 (4)	0.4724 (2)	0.0367 (7)	
C9	0.3296 (4)	−0.0471 (4)	0.3930 (2)	0.0458 (9)	
H9A	0.3288	−0.1616	0.3996	0.055*	
C10	0.3893 (4)	0.0400 (4)	0.3058 (2)	0.0463 (9)	
H10A	0.4292	−0.0178	0.2550	0.056*	
C11	0.3924 (4)	0.2150 (4)	0.2904 (2)	0.0408 (8)	
C12	0.3347 (4)	0.2909 (4)	0.3723 (2)	0.0411 (8)	
C13	0.2751 (4)	0.2014 (4)	0.4597 (2)	0.0417 (8)	
H13A	0.2376	0.2568	0.5116	0.050*	
C14	0.5150 (5)	0.2322 (4)	0.1184 (2)	0.0422 (8)	
C15	0.6711 (5)	0.0901 (5)	0.1283 (2)	0.0518 (9)	
H15A	0.7234	0.0318	0.1914	0.062*	
C16	0.7504 (5)	0.0333 (5)	0.0459 (3)	0.0574 (10)	
H16A	0.8563	−0.0623	0.0524	0.069*	
C17	0.6702 (6)	0.1204 (5)	−0.0458 (3)	0.0554 (10)	
C18	0.5142 (5)	0.2600 (5)	−0.0589 (2)	0.0566 (10)	
H18A	0.4617	0.3155	−0.1221	0.068*	
C19	0.4358 (5)	0.3171 (4)	0.0244 (2)	0.0495 (9)	
H19A	0.3298	0.4126	0.0173	0.059*	

### Atomic displacement parameters (Å^2^)

**Table:**

	*U*^11^	*U*^22^	*U*^33^	*U*^12^	*U*^13^	*U*^23^
F1	0.114 (2)	0.0861 (17)	0.0570 (13)	−0.0353 (15)	0.0258 (13)	−0.0374 (12)
N1	0.065 (2)	0.0442 (16)	0.0382 (15)	−0.0275 (15)	0.0133 (13)	−0.0135 (12)
F2	0.108 (2)	0.0535 (14)	0.0715 (14)	−0.0251 (13)	0.0345 (13)	0.0043 (11)
C1	0.094 (3)	0.038 (2)	0.068 (3)	−0.020 (2)	0.017 (2)	−0.0092 (19)
N2	0.078 (2)	0.0423 (16)	0.0389 (16)	−0.0291 (16)	0.0172 (15)	−0.0130 (13)
O1	0.104 (2)	0.0492 (15)	0.0487 (14)	−0.0345 (15)	0.0227 (14)	−0.0192 (12)
C2	0.090 (3)	0.044 (2)	0.050 (2)	−0.024 (2)	0.020 (2)	−0.0121 (17)
O2	0.136 (3)	0.0554 (16)	0.0587 (16)	−0.0535 (17)	0.0343 (16)	−0.0184 (13)
C3	0.052 (2)	0.042 (2)	0.0342 (18)	−0.0201 (17)	0.0066 (16)	−0.0069 (15)
N3	0.089 (3)	0.0494 (18)	0.0465 (18)	−0.0370 (18)	0.0176 (17)	−0.0165 (16)
C4	0.063 (3)	0.055 (2)	0.044 (2)	−0.031 (2)	0.0094 (18)	−0.0129 (17)
O3	0.225 (4)	0.072 (2)	0.0626 (18)	−0.087 (2)	0.050 (2)	−0.0403 (16)
C5	0.068 (3)	0.064 (3)	0.043 (2)	−0.028 (2)	0.0144 (18)	−0.0145 (18)
C6	0.062 (3)	0.046 (2)	0.049 (2)	−0.0125 (19)	0.0141 (18)	0.0043 (17)
C7	0.045 (2)	0.0400 (19)	0.0396 (18)	−0.0162 (17)	0.0017 (16)	−0.0091 (16)
C8	0.040 (2)	0.0355 (18)	0.0351 (17)	−0.0139 (15)	−0.0003 (14)	−0.0072 (14)
C9	0.061 (2)	0.043 (2)	0.0426 (19)	−0.0269 (18)	0.0063 (17)	−0.0134 (15)
C10	0.060 (2)	0.046 (2)	0.0427 (19)	−0.0260 (18)	0.0105 (17)	−0.0201 (16)
C11	0.044 (2)	0.0385 (19)	0.0412 (19)	−0.0149 (16)	0.0005 (15)	−0.0106 (15)
C12	0.053 (2)	0.0365 (18)	0.0384 (18)	−0.0195 (17)	0.0029 (16)	−0.0110 (15)
C13	0.053 (2)	0.0418 (19)	0.0339 (17)	−0.0186 (17)	0.0030 (15)	−0.0116 (14)
C14	0.060 (2)	0.0416 (19)	0.0353 (18)	−0.0296 (19)	0.0097 (16)	−0.0125 (15)
C15	0.062 (3)	0.050 (2)	0.043 (2)	−0.020 (2)	0.0041 (18)	−0.0094 (16)
C16	0.064 (3)	0.049 (2)	0.060 (2)	−0.0173 (19)	0.014 (2)	−0.0197 (19)
C17	0.082 (3)	0.060 (2)	0.041 (2)	−0.040 (2)	0.022 (2)	−0.0237 (18)
C18	0.073 (3)	0.065 (3)	0.039 (2)	−0.036 (2)	0.0023 (19)	−0.0083 (18)
C19	0.050 (2)	0.046 (2)	0.049 (2)	−0.0173 (18)	0.0048 (18)	−0.0066 (17)

### Geometric parameters (Å, °)

**Table:**

F1—C17	1.358 (3)	C5—H5A	0.9300
N1—C7	1.365 (4)	C7—C8	1.490 (4)
N1—C3	1.413 (3)	C8—C13	1.376 (4)
N1—H1A	0.8600	C8—C9	1.400 (4)
F2—C6	1.373 (4)	C9—C10	1.369 (4)
C1—C6	1.354 (5)	C9—H9A	0.9300
C1—C2	1.382 (4)	C10—C11	1.415 (4)
C1—H1C	0.9300	C10—H10A	0.9300
N2—C11	1.357 (4)	C11—C12	1.412 (4)
N2—C14	1.421 (4)	C12—C13	1.378 (4)
N2—H2A	0.8600	C13—H13A	0.9300
O1—C7	1.225 (3)	C14—C15	1.376 (4)
C2—C3	1.379 (4)	C14—C19	1.388 (4)
C2—H2B	0.9300	C15—C16	1.375 (4)
O2—N3	1.222 (3)	C15—H15A	0.9300
C3—C4	1.380 (4)	C16—C17	1.366 (5)
N3—O3	1.214 (3)	C16—H16A	0.9300
N3—C12	1.451 (4)	C17—C18	1.363 (5)
C4—C5	1.381 (4)	C18—C19	1.384 (4)
C4—H4A	0.9300	C18—H18A	0.9300
C5—C6	1.360 (5)	C19—H19A	0.9300
			
C7—N1—C3	128.1 (3)	C10—C9—C8	121.5 (3)
C7—N1—H1A	115.9	C10—C9—H9A	119.3
C3—N1—H1A	115.9	C8—C9—H9A	119.3
C6—C1—C2	117.9 (4)	C9—C10—C11	122.1 (3)
C6—C1—H1C	121.0	C9—C10—H10A	118.9
C2—C1—H1C	121.0	C11—C10—H10A	118.9
C11—N2—C14	126.9 (3)	N2—C11—C12	123.5 (3)
C11—N2—H2A	116.6	N2—C11—C10	121.2 (3)
C14—N2—H2A	116.6	C12—C11—C10	115.2 (3)
C3—C2—C1	121.4 (3)	C13—C12—C11	121.9 (3)
C3—C2—H2B	119.3	C13—C12—N3	116.8 (3)
C1—C2—H2B	119.3	C11—C12—N3	121.2 (3)
C2—C3—C4	118.6 (3)	C8—C13—C12	121.9 (3)
C2—C3—N1	117.8 (3)	C8—C13—H13A	119.0
C4—C3—N1	123.6 (3)	C12—C13—H13A	119.0
O3—N3—O2	120.7 (3)	C15—C14—C19	119.5 (3)
O3—N3—C12	118.4 (3)	C15—C14—N2	121.5 (3)
O2—N3—C12	120.9 (3)	C19—C14—N2	118.8 (3)
C3—C4—C5	120.5 (3)	C16—C15—C14	120.6 (3)
C3—C4—H4A	119.7	C16—C15—H15A	119.7
C5—C4—H4A	119.7	C14—C15—H15A	119.7
C6—C5—C4	118.7 (3)	C17—C16—C15	118.5 (4)
C6—C5—H5A	120.7	C17—C16—H16A	120.7
C4—C5—H5A	120.7	C15—C16—H16A	120.7
C1—C6—C5	122.9 (3)	F1—C17—C18	119.1 (3)
C1—C6—F2	119.1 (3)	F1—C17—C16	118.1 (4)
C5—C6—F2	117.9 (3)	C18—C17—C16	122.8 (3)
O1—C7—N1	122.2 (3)	C17—C18—C19	118.3 (3)
O1—C7—C8	120.9 (3)	C17—C18—H18A	120.8
N1—C7—C8	116.9 (3)	C19—C18—H18A	120.8
C13—C8—C9	117.3 (3)	C18—C19—C14	120.2 (3)
C13—C8—C7	115.9 (3)	C18—C19—H19A	119.9
C9—C8—C7	126.8 (3)	C14—C19—H19A	119.9
			
C6—C1—C2—C3	−0.9 (6)	N2—C11—C12—C13	175.3 (3)
C1—C2—C3—C4	0.5 (6)	C10—C11—C12—C13	−1.7 (5)
C1—C2—C3—N1	−178.1 (3)	N2—C11—C12—N3	−5.3 (5)
C7—N1—C3—C2	178.7 (3)	C10—C11—C12—N3	177.6 (3)
C7—N1—C3—C4	0.2 (5)	O3—N3—C12—C13	7.8 (5)
C2—C3—C4—C5	0.4 (5)	O2—N3—C12—C13	−172.6 (3)
N1—C3—C4—C5	178.9 (3)	O3—N3—C12—C11	−171.5 (4)
C3—C4—C5—C6	−0.9 (5)	O2—N3—C12—C11	8.1 (5)
C2—C1—C6—C5	0.5 (6)	C9—C8—C13—C12	0.8 (5)
C2—C1—C6—F2	178.4 (3)	C7—C8—C13—C12	−179.1 (3)
C4—C5—C6—C1	0.4 (6)	C11—C12—C13—C8	0.4 (5)
C4—C5—C6—F2	−177.5 (3)	N3—C12—C13—C8	−178.9 (3)
C3—N1—C7—O1	0.3 (5)	C11—N2—C14—C15	−56.2 (5)
C3—N1—C7—C8	−179.6 (3)	C11—N2—C14—C19	128.7 (3)
O1—C7—C8—C13	1.9 (5)	C19—C14—C15—C16	1.0 (5)
N1—C7—C8—C13	−178.2 (3)	N2—C14—C15—C16	−174.0 (3)
O1—C7—C8—C9	−178.0 (3)	C14—C15—C16—C17	−0.4 (5)
N1—C7—C8—C9	1.9 (5)	C15—C16—C17—F1	178.9 (3)
C13—C8—C9—C10	−0.7 (5)	C15—C16—C17—C18	−0.6 (5)
C7—C8—C9—C10	179.2 (3)	F1—C17—C18—C19	−178.4 (3)
C8—C9—C10—C11	−0.6 (5)	C16—C17—C18—C19	1.0 (5)
C14—N2—C11—C12	173.7 (3)	C17—C18—C19—C14	−0.4 (5)
C14—N2—C11—C10	−9.4 (5)	C15—C14—C19—C18	−0.5 (5)
C9—C10—C11—N2	−175.3 (3)	N2—C14—C19—C18	174.6 (3)
C9—C10—C11—C12	1.8 (5)		

### Hydrogen-bond geometry (Å, °)

**Table:**

*D*—H···*A*	*D*—H	H···*A*	*D*···*A*	*D*—H···*A*
N1—H1A···O3^i^	0.86	2.37	3.185 (4)	158
N2—H2A···O2	0.86	1.98	2.636 (4)	132
N2—H2A···N3	0.86	2.58	2.917 (4)	105
C2—H2B···O3^i^	0.93	2.40	3.240 (5)	151
C4—H4A···O1	0.93	2.20	2.821 (4)	123
C10—H10A···F1^ii^	0.93	2.53	3.205 (4)	130
C13—H13A···O1	0.93	2.39	2.728 (4)	102
C13—H13A···O3	0.93	2.33	2.661 (5)	100
C15—H15A···O1^iii^	0.93	2.55	3.454 (4)	164
C16—H16A···F2^iv^	0.93	2.39	3.272 (5)	158

Symmetry codes: (i) *x*, *y*−1, *z*; (ii) −*x*+1, −*y*, −*z*; (iii) −*x*+1, −*y*, −*z*+1; (iv) −*x*+1, −*y*−1, −*z*+1.
